# Supplementary figures and images for: Allogeneic testes transplanted into partially castrated adult medaka (Oryzias latipes) can produce donor-derived offspring by natural mating over a prolonged period
Source: Zoological Lett. 2022 Jul 25;8:10. doi: 10.1186/s40851-022-00195-1 (PMC9310406; doi:10.1186/s40851-022-00195-1)

a

*actb*-GFP hetero

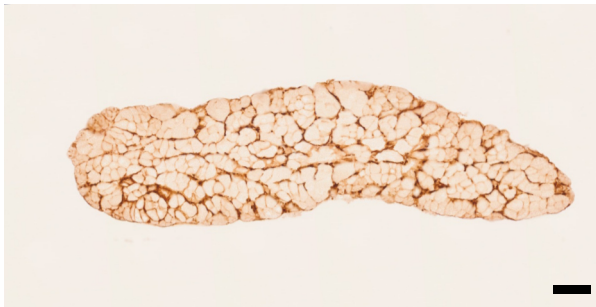

d-rR

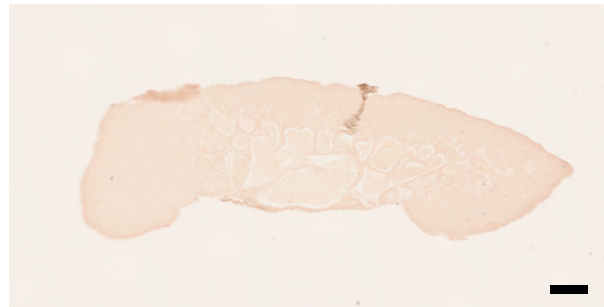

b

*actb*-GFP hetero

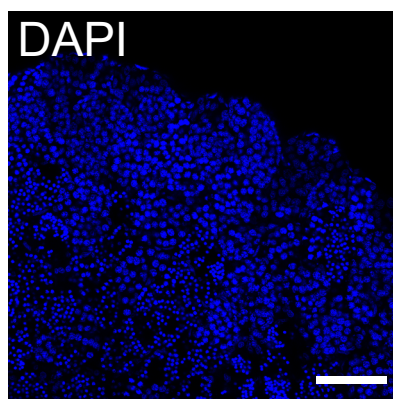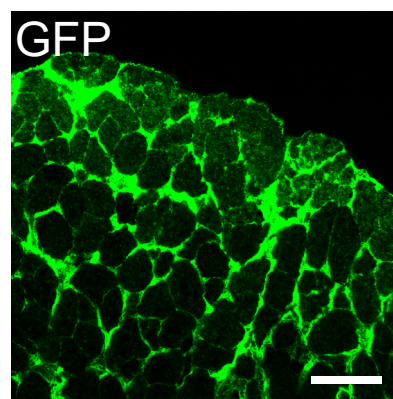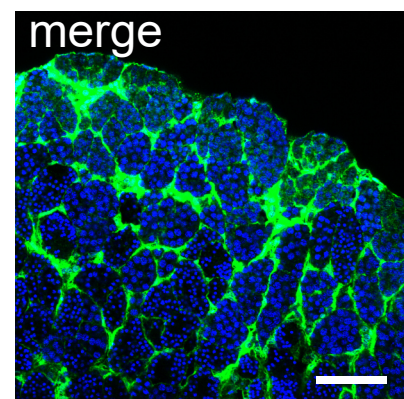

d-rR

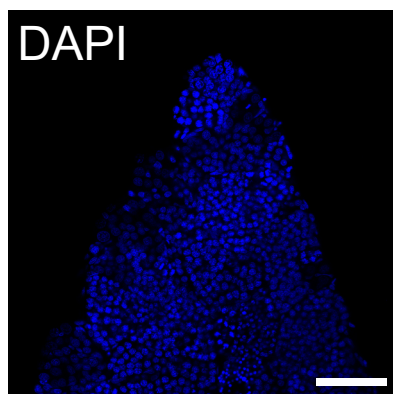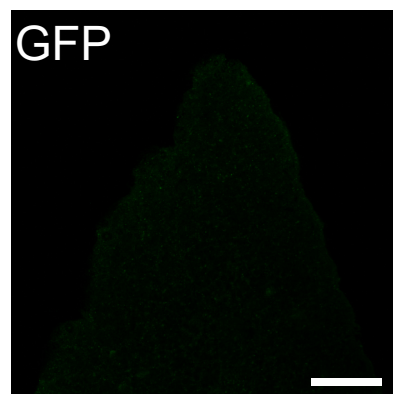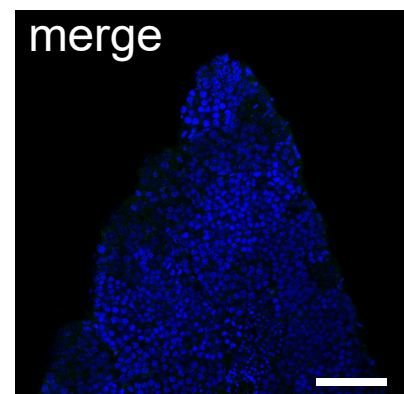

Supplement: Supplementary file 1 — Additional file1: Figure S1. The protein level of GFP in the germ cells is relatively low compared to that of the surrounding somatic cells. (a, b) Representative images from the IHC analysis using an anti-GFP antibody visualized by DAB staining (a) or fluorescent detection (b). (a) Left panel shows an image of the testis that consistently expressed GFP with beta-actin (actb-GFP hetero). Right panel shows an image of the testis of d-rR (recipient) strain. Scale bar, 100 μm. (b) Upper and lower panels show an image of actb-GFP hetero and d-rR testis, respectively. Left and middle panels show images of DAPI (blue) and GFP (green), respectively, in the same section; right panel shows the merged image. The GFP signal in germ cells was faint in the fluorescent observation. Scale bar, 50 μm. [file 40851_2022_195_MOESM1_ESM.pdf]
